# The Unexpected High Solubility of Fluorinated Zinc Phthalocyanines in Aqueous Solutions and Their Use for the Preparation of Photodynamic Coatings on Various Substrates

**DOI:** 10.1021/acs.langmuir.4c05325

**Published:** 2025-03-20

**Authors:** Jonathan Pinnock, Kai Hansen, Marius Pelmuş, Alexander Y. Fadeev

**Affiliations:** † Department of Chemistry and Biochemistry, Seton Hall University, South Orange, New Jersey 07079, United States; ‡ Center for Functional Materials, Seton Hall University, South Orange, New Jersey 07079, United States

## Abstract

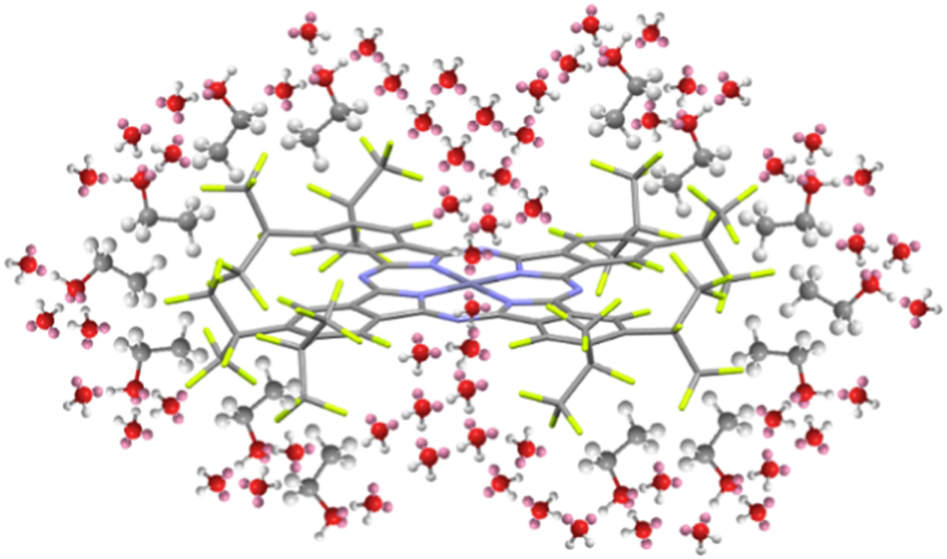

We report the results of the investigation of aqueous solutions of perfluorinated metal phthalocyanines F*_x_*PcZn (*x* = 16, 64) and their adsorption at solid–liquid interfaces. Specifically, we focused on the development of environmentally benign methods for the preparation of coatings of fluorinated photosensitizers that are capable of generating singlet oxygen and degrading organic contaminants in water. Counterintuitively, F_16_PcZn and F_64_PcZn demonstrated good solubility (∼10^–4^ M) in water/ethanol mixtures containing up to 95–98% water by volume. The amphiphilic properties of the perfluorinated macrocycles were attributed to the presence of a highly polar metal porphyrin center surrounded by the nonpolar peripheral groups (F and CF_3_). A thermodynamic model of the interfacial interactions upon solvation of the amphiphilic phthalocyanines was proposed, rationalizing their solubility in water–ethanol. As assessed by UV–vis and fluorescence, aqueous solution of F_16_ and F_64_PcZn contained aggregates; however, the size of the aggregates was small and not detectable by DLS. The adsorption of phthalocyanines from the water–ethanol mixtures occurred readily on various substrates, including PET plastic, polyester and nylon textiles, mica, silica gel, and talc. According to XPS, solid-state UV–vis, and contact angles, the adsorption from water–ethanol solutions produced hydrophobic, ∼monomolecular coatings of the phthalocyanines. As compared to solutions, the fraction of the monomeric phthalocyanines significantly increased in the adsorbed state. The shift of the self-association equilibrium toward monomers was particularly notable for the adsorption on talc, where both phthalocyanines were monomeric even after the adsorption from solutions with high water content. The photodynamic activity of the coatings was tested in the reactions of degradation of methyl orange dye under radiation with visible light. Both F_16_PcZn and F_64_PcZn adsorbed on talc produced active, stable, and reproducible coatings capable of degrading methyl orange dye in water.

## Introduction

Phthalocyanines are purely synthetic highly conjugated macrocycle derivatives from the porphyrinoids family that traditionally are being used as dyes and pigments (paints, coatings, printing inks, tattoos) and recently in hi-tech applications such as photodynamic therapy and imaging, optical data storage, reverse saturable absorbers, solar screens, and electrocatalysis.^1,2^

Phthalocyanines and their metal complexes show notoriously low solubility^3−5^ in water and common organic solvents, limiting their applications in wet technologies, e.g., in preparing coatings, sensors, catalysts, etc. Several synthetic strategies have been explored to overcome the poor solubility of the phthalocyanines. The main approach revolves around synthesizing the phthalocyanines decorated with bulky lipophilic or hydrophilic substituents^6,7^ aiming to reduce their aggregation and enhance their interactions with solvent. In photodynamic therapy and bioimaging,^8^ where water solubility is essential, phthalocyanines containing polar or ionic substituents can be used directly or in conjugation with water-soluble polymers, nanoparticles, micelles, and liposomes.^9^

Fluorination of phthalocyanines^10,11^ improves their solubility in organic solvents with medium polarity (tetrahydrofuran, ethyl acetate, acetone, ethanol) compared to the nonfluorinated counterparts. The electron-withdrawing effect of the fluorine atoms and perfluorinated substituents enhances the activity of the molecule in the generation of singlet oxygen,^12,13^ which makes these molecules promising for applications in photodynamic coatings and surfaces. Additionally, C–F bonds resist oxidation^14^ thereby improving the stability of the molecules in the presence of reactive oxygen species, e.g., under the conditions of oxidation photocatalysis. The introduction of bulky perfluoroalkyl substitutes, e.g., perfluoro isopropyl, further improves solubility and additionally reduces the ability of phthalocyanines to aggregate by hindering the π–π stacking interactions.^15^

Aqueous solutions of fluorinated phthalocyanines and their interactions with water remain largely an unexplored field. The only account in the literature was the original work,^16^ in which the authors reported “surprising”, and “very limited solubility” of F_64_PcH_2_ and F_64_PcZn in aqueous solutions. We found no references regarding the solubility of F_16_PcZn in aqueous media.

In our previous work,^17^ we reported large adsorption of water vapors on powders of perfluorinated zinc phthalocyanines, an unexpected finding for what was believed to be hydrophobic compounds. This prompted our interest in exploring the amphiphilic properties of these molecules further and to study their solutions in water-organic mixtures. In the work presented here, we report the preparation of aqueous solutions of F*_x_*PcZn (*x* = 16, 64) and their use for the deposition of thin phthalocyanine films on various polymer and mineral substrates, including polyester and nylon textiles, PET plastic, mica, silica gel, and talc. The surfaces were characterized by UV–vis, XPS, and contact angles, and their photodynamic activity was tested in the reaction of photodegradation of the methyl orange dye in water.

## Experimental Section

### Chemicals and Substrates

**Figure 1:**
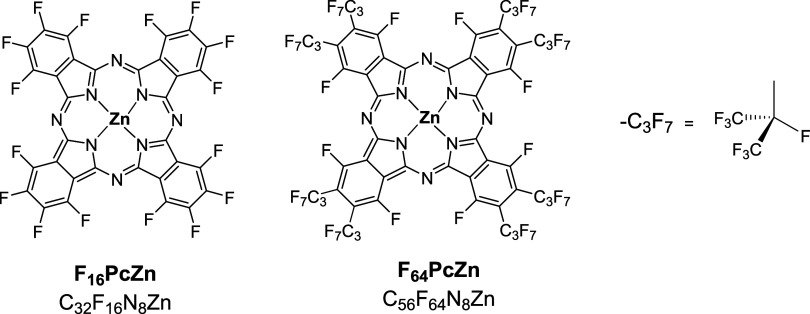
Chemical structures of the studied phthalocyanines.

Solvents were obtained from Sigma-Aldrich and used without further purification, unless stated otherwise. The fluorinated zinc phthalocyanines F_16_PcZn and F_64_PcZn (Figure 1) were synthesized by tetramerization of the corresponding phthalonitriles in the presence of zinc acetate under microwave radiation.^18^ Samples of polyester and nylon textiles were a kind gift from Dr. Heather Hays (Milliken, Inc.) and used as provided. The poly(ethylene terephthalate) (PET) plastic samples were cut from the transparency films by 3 M, Inc. To clean, the PET samples were rinsed with soapy water and then with deionized water. The samples of mica were cut and freshly peeled from high-grade mica sheets (MTI, Inc.). Mesoporous silica gel, 200–400 mesh, *S*_BET_(N_2_) = 200 m^2^/g (Sigma-Aldrich, Inc.) was used as received. Talc (Luzenac, Inc.), particle size 1.7 μm, *S*_BET_(N_2_) = 14 m^2^/g was a kind gift from Dr. Misha Gelfer (SUNY, Stony Brook).

### Solutions of Phthalocyanines

The solutions of phthalocyanines in pure ethanol and water–ethanol mixtures were prepared from the corresponding stock solutions via sequential dilution with pure ethanol or water–ethanol mixtures. According to the literature,^19^ water–ethanol mixtures form stable microscopic bubbles in the range of ∼ 100 nm that can impact the UV–vis and, especially, the light scattering measurements. To remove the bubbles, the water−ethanol mixtures were filtered twice through a 0.2 μm Teflon membrane prior to dissolution of the phthalocyanines. Dynamic Light Scattering (DLS) of the solutions was performed using a NanoBrook 90Plus particle sizer. According to DLS, the prepared water–ethanol mixtures contained no scattering particles greater than 0.5 nm, i.e., were free from nanobubbles. The UV–vis spectra of solutions were recorded with a Hewlett-Packard Diode Array Spectrophotometer (8452A) and with a Vernier Go Direct SpectroVis Plus instrument. The fluorescence spectra were obtained using a HORIBA Fluorolog-3 Spectrofluorometer (Model FL-1057). The spectra were measured for the solutions of F_64_PcZn in water–ethanol with and without addition of Twin 20. The excitation wavelength was 650 nm, and the bandwidth was 5 nm. Solubility of the phthalocyanines was determined by UV–vis absorbance of the saturated water–ethanol solutions using the molar extinction presented in Table 1. The reported values of solubility were the average of duplicate measurements.

**Table 1:** UV–vis Characteristics for the F_16_PcZn and F_64_PcZn in Water–Ethanol Solutions

	**F**_**16**_**PcZn**			**F**_**64**_**PcZn**		
**solvent** (vol %)	**λ**_**max**_	**log ε**	**fwhm, nm**	**λ**_**max**_	**log ε**	**fwhm, nm**
pure ethanol	638	4.84	44	684	5.29	24
water–ethanol (50–50)	645	4.60	52	688	5.19	43
water–ethanol (75–25)	648	4.45	80	690	5.09	45
water–ethanol (90–10)	649	4.36	83	692	4.94	46
water–ethanol (95–5)	649	4.23	88	692	4.61	46

### Preparation and Characterization of Coatings

A sample of the substrate (∼1 cm × 1 cm piece of textile, PET plastic, mica sheet, 250 mg of silica gel or talc) was placed in a vial containing 5 mL of a solution of phthalocyanine of specific concentration (1–200 μM). The vials were capped and left overnight in the dark. The substrate was removed from the solution using tweezers (for single surfaces) or transferred on a glass filter (for powders). The excess liquid was shaken off or removed by filtration, and then the sample was dried in an oven at 60 °C for 30 min. The solid-state electronic spectra were collected using a Shimadzu 3600 plus UV–vis-NIR spectrometer equipped with a diffuse-reflectance accessory. X-ray photoelectron spectroscopy (XPS) was done using a Thermo Scientific spectrometer, an Al Kα microfocused monochromatized source (1486.6 eV) with a resolution of 0.6 eV. The spot size was 400 μm, and the operating pressure was 5 × 10^–9^ Pa. Contact angle measurements were performed on a Kyowa DMo-502 goniometer using reverse osmosis purified water and hexadecane as the probe fluids. Dynamic advancing (θ_A_) and receding angles (θ_R_) were recorded while the probe fluid was added to and withdrawn from the drop, respectively. The average of 3–5 measurements is reported; the error of the measurements was 1–2°.

### Photochemical Degradation of Methyl Orange Dye

The photochemical activity of F_16_PcZn and F_64_PcZn coatings was tested in the photodegradation of methyl orange (MO) dye using the following general protocol. 10 mL of 15 μM solution of MO in DI water was placed in a Petri dish that contained a sample of catalyst (polyester textile, cut in a round shape ∼25 mm in diameter or 250 mg of talc). A Petri dish was placed in a thermostatic jacketed reactor at 25 °C. Illumination of 120,000 lux intensity was applied using Kodak projector with a 300 W halogen lamp. The intensity of the light was measured with Extech light meter. Aliquots of solution were taken hourly over 4–10 h time periods, and the solution concentration of MO was determined by UV–vis absorbance at 464 nm (ε = 21,600 M^–1^ cm^–1^).

## Results and Discussion

### Amphiphilic Nature of Fluorinated Phthalocyanines and Properties of Their Aqueous Solutions

**Figure 2:**
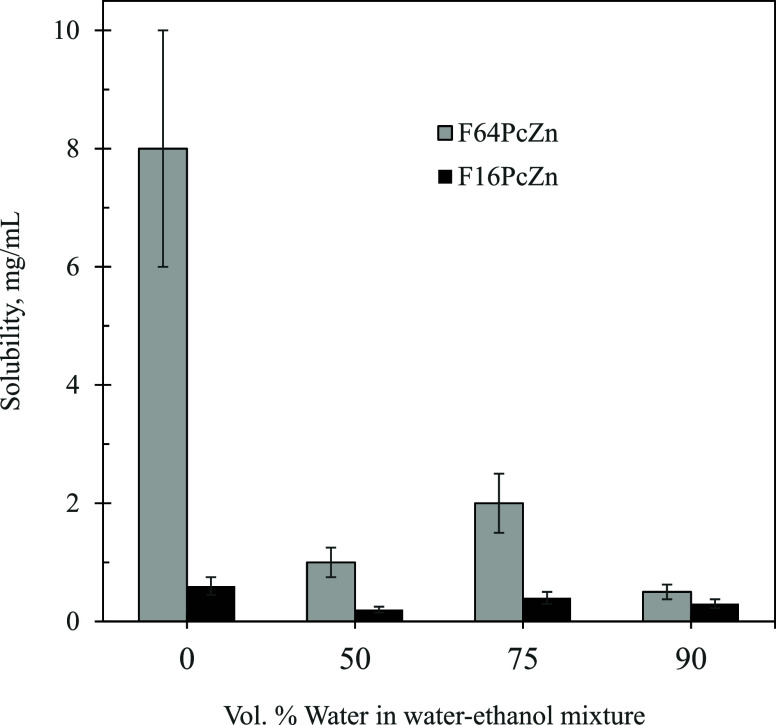
Solubility of F_64_PcZn and F_16_PcZn in water–ethanol mixtures.

Remarkably, F_16_PcZn containing 35 mass % fluorine and F_64_PcZn containing 59 mass % fluorine (Figure 1) demonstrated relatively good solubility in water–ethanol mixtures. The solubility of phthalocyanines in pure ethanol was ∼0.6 mg/mL (∼7 × 10^–4^ M) for F_16_PcZn and ∼10 mg/mL (∼5 × 10^–3^ M) for F_64_PcZn, respectively. In solutions containing 90 vol % of water, F_16_PcZn was soluble at the level ∼0.3 mg/L (∼4 × 10^–4^ M) and F_64_PcZn ∼1 mg/mL (∼5 × 10^–4^ M), respectively, Figure 2.

**Figure 3:**
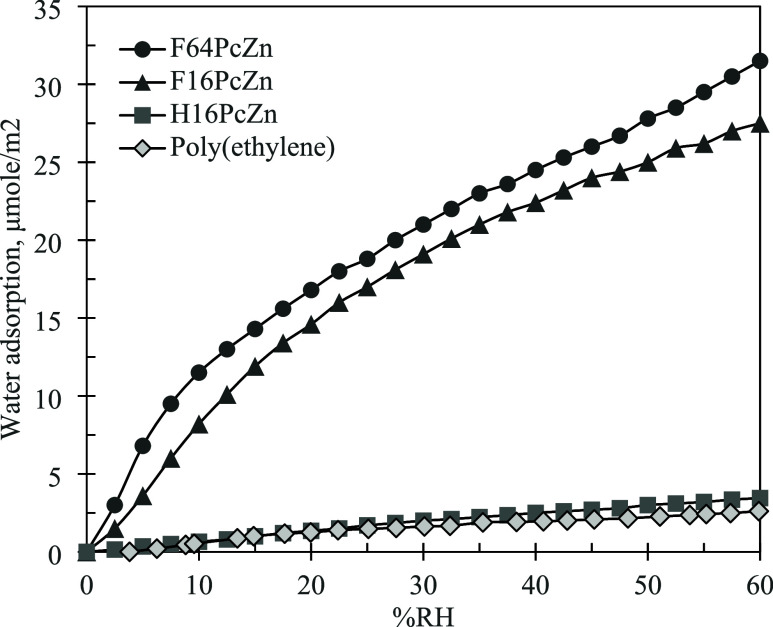
Water vapor adsorption isotherms (295 K) on phthalocyanine powders and on poly(ethylene).

The fluorinated extremities of the macrocycles were nonpolar and hydrophobic, so the overall solubility of the compounds in aqueous media was indicative of the presence of the strong polar site(s) capable of interactions with water, i.e., their amphiphilic nature. The central metal can play the role of a polar center. Due to the presence of strong electron-withdrawing groups (F and CF_3_) surrounding the macrocycles, the central metal ion Zn^2+^ experienced electron deficiency resulting in the formation of a strong polar center (Lewis acid) capable of coordinating water molecules. In our previous work,^17^ the high affinity of F_16_ and F_64_PcZn to water was demonstrated through the water adsorption experiments. Figure 3 compares the water vapor equilibrium adsorption isotherms for the powders of neat F_16_ and F_64_PcZn. The data for unsubstituted H_16_PcZn and for poly(ethylene) are also included for comparison. The isotherms are presented as μmol of adsorbed water per m^2^ of the adsorbing surfaces. This enables a direct comparison of the adsorption for different surfaces by accounting for the differences in the powder’s particle size and surface area. As shown in Figure 3, the adsorption of water on H_16_PcZn was very low, similar to the adsorption of water on poly(ethylene), a typical nonpolar, hydrophobic surface. For F_16_PcZn and F_64_PcZn, the adsorption of water increased ∼10-fold vs H_16_PcZn over the entire range of pressures demonstrating the presence of strong polar centers in F_16_PcZn and F_64_PcZn.

To rationalize the solubility of the fluorinated zinc phthalocyanines in aqueous solutions, we considered the free energy change for a process of transfer of the phthalocyanine from a vacuum to a water–ethanol solution. This process was accompanied by (i) an increase in free energy due to the insertion of a hydrophobic molecule in aqueous media (Δ*G*_nonpolar_ > 0) and (ii) a decrease in free energy due to the interactions of the polar center of the phthalocyanine with the solvent (Δ*G*_polar_ < 0). To determine the contributions of Δ*G*_polar_ and Δ*G*_nonpolar_ and the net Δ*G* of the process, the surface area of the fluorinated zinc phthalocyanine molecules was treated as a superposition of *A*_polar_ and *A*_nonpolar_. *A*_polar_ included azo-bridged pyrroles and the Zn atom. *A*_nonpolar_ included benzene rings with aromatic and aliphatic fluorines, as shown in Figure 1. Δ*G*_nonpolar_ was evaluated from the interfacial tension at the solid–liquid interface using the literature data on wetting of poly(tetrafluoroethylene) by water–ethanol mixtures.^RefS3^ Δ*G*_polar_ was evaluated from the data on free energy of immersion^RefS5^ of polar mineral surfaces in water and ethanol. The details of the calculations are provided in the Supplement, Tables S1 and S2. The results showed that even for the solutions containing 95 vol % of water, the absolute value of Δ*G*_polar_ was greater than Δ*G*_nonpolar_ demonstrating that the gain from the specific interactions between water and the polar center of the molecules compensated for the free energy loss due to the formation of a hydrophobic interface. As both phthalocyanines were practically insoluble in pure water, the role of ethanol in their solubility was critical. It was known in the literature that organic cosolvents significantly improved the solubility of bulk aromatic hydrocarbons such as PAHs in water.^20^ Similar to the observation made by the authors^20^ for their systems, the solubility of the phthalocyanines in this work was not much influenced by the amount of water in solutions with high water content (Figure 2). We attributed this to the “effect of saturation” by ethanol, which was present in a large molar excess compared to phthalocyanines. For example in a 100 μM solution of phthalocyanine in 90–10 vol % water–ethanol, the molar fraction of ethanol was 3 × 10^–2^ vs 4 × 10^–6^ for the phthalocyanine. The large excess of ethanol reduced the interfacial tension and facilitated the solvation of nonpolar portions of the phthalocyanines. We noted that the simple model presented above provided only an estimate of the energies involved and qualitatively explained the solubility of fluorinated metal phthalocyanines in water-organic solutions. More thorough treatment should account for the aggregation of molecules in solution, which was evident from the spectral data described in the next sections.

**Figure 4:**
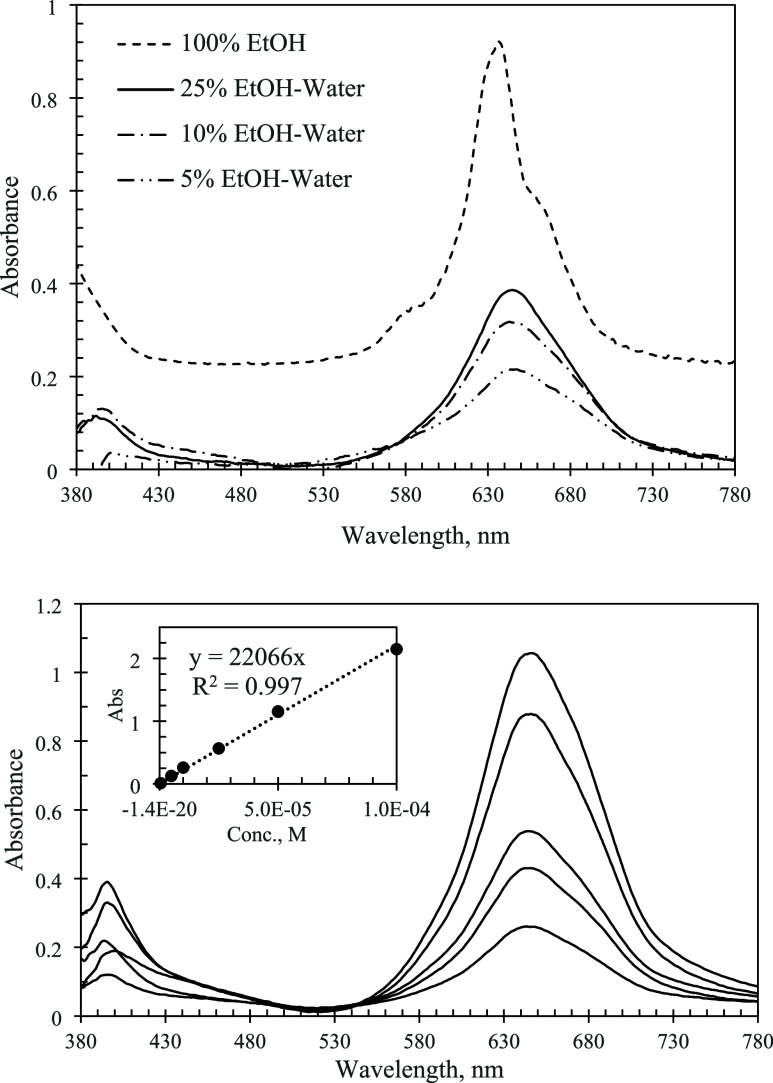
Top: Electronic absorption spectra of F_16_PcZn in water–ethanol mixtures of different compositions. Concentration of phthalocyanine in all -15 μM. The spectrum in 100% ethanol is offset for clarity. Bottom: Spectra of 45, 34, 24, 19, and 10 μM solutions of F_16_PcZn in 90–10 vol % water–ethanol. Inset: Beer–Lambert plot for the Q-band.

The state of the fluorinated phthalocyanines in solution was monitored by UV–vis spectroscopy. The electronic absorbance spectra of the solutions of F_16_PcZn are shown in Figure 4. Like many metal phthalocyanines that lack bulky peripheral substitutes, F_16_PcZn is prone to aggregation due to π-π stacking.^21,22^ In ethanol, the aggregates of F_16_PcZn were favored. This was demonstrated by the dominating “aggregated” band with a peak maximum of ∼ 640 nm and a shoulder of ∼ 670 nm from the “monomer”. In aqueous solutions, the spectra showed major changes that were indicative of the progressive aggregation of the molecules. As shown in Figure 4, for water-organic solutions, the Q-band was broadened and its intensity was decreased significantly. The molar extinction in 95–5 vol % water–ethanol dropped more than 4 times versus that in pure ethanol. The maximum wavelength (λ_max_) also demonstrated large blue shifts reflecting the changes in the local molecular environment, Table 1. To check whether the aggregation equilibria were affected by the solution concentrations, the spectra were recorded at different concentrations. A representative example of the spectra and the corresponding Beer–Lambert plot are shown at the bottom of Figure 4. Adequate linear fits of the Lambert–Beer plots for the Q bands were observed for all of the solutions of F_16_PcZn in the concentration range 1 to 100 μM. This led us to conclude that, in the concentration range studied, the aggregation equilibria were not affected by the concentration of solutions.

**Figure 5:**
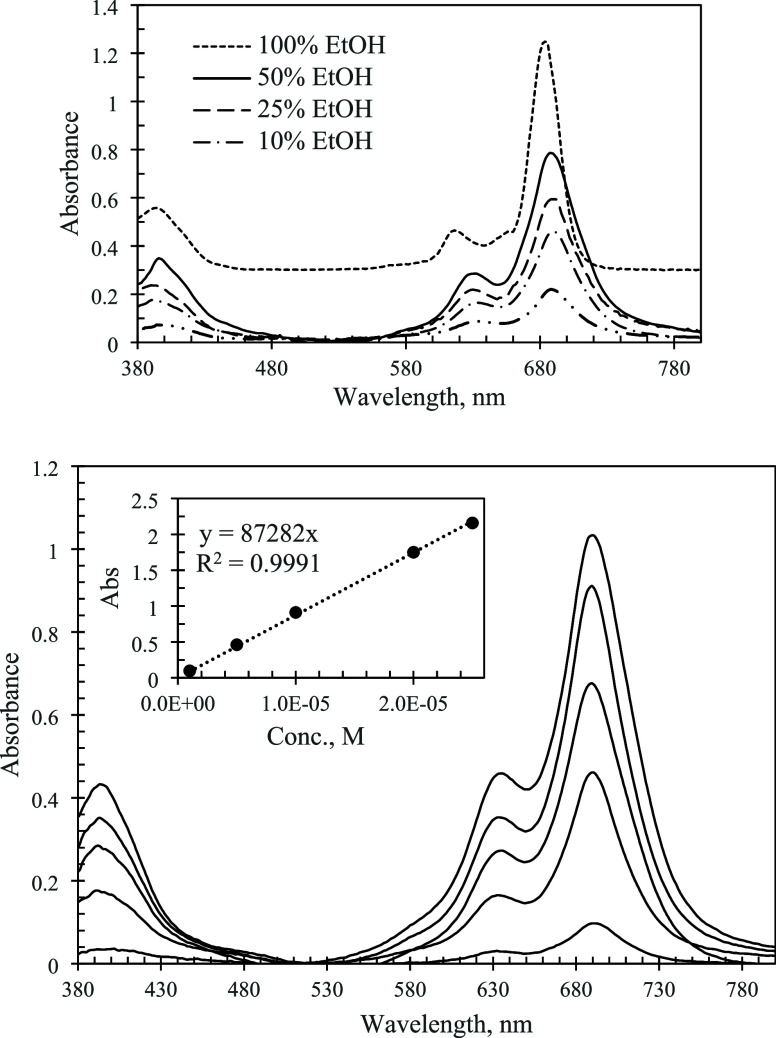
Top: Electronic absorption spectra of F_64_PcZn in water–ethanol mixtures of different compositions. Concentration of phthalocyanine in all -5 μM. The spectrum in 100% ethanol is offset for clarity. Bottom: Spectra of 12.5, 9, 7, 5, and 1 μM solutions of F_64_PcZn in 90–10 vol % water–ethanol mixture. Inset: Beer–Lambert plot for the Q-band.

The electronic spectra of F_64_PcZn are shown in Figure 5. In pure ethanol, the spectra demonstrated a sharp, intense Q-band at 684 nm with an associated higher-energy vibrational component at 618 nm. This was characteristic of a monomeric, nonaggregated state of the phthalocyanine in solution.^15,16^ The resistance of F_64_PcZn to aggregate in organic solutions was attributed to the presence of the 8 bulky perfluoroisopropyl groups decorating the molecule and thus preventing the stacking. In water−ethanol solutions of F_64_PcZn, a new absorbance band, which was not present in pure ethanol, appeared on the lower-wavelength side of the Q-band. The peak maxima shifted to a higher wavelength, and the spectra became significantly broader, Figure 5. The molar extinction in 95–5 vol % water–ethanol dropped almost 5 times vs that in pure ethanol, Table 1. These changes were attributed to the formation of aggregates of F_64_PcZn. The relative intensity of the aggregates (∼636 nm) vs the monomers (∼690 nm) increased with the solution concentration of F_64_PcZn indicating an increase in the aggregation. The aggregation of F_64_PcZn was also evident from the strong decrease in the fluorescence intensity for the solutions containing more than 70 vol % of water. Spiking water solutions with Twin 20 restored the fluorescence output (Figure S1) demonstrating that the aggregation was reversible. The electronic spectra of the F_16_PcZn and F_64_PcZn water–ethanol solutions did not change over six months indicating a high chemical stability of the phthalocyanines, even at 95 and 98 vol % of water.

To assess the size of the phthalocyanine aggregates, the solutions have been investigated using the dynamic light scattering technique (DLS). Surprisingly, it was determined that the size of the scattering particles showed almost no variation (within the error of the method) in the solution composition for both F_64_PcZn and F_16_PcZn. Regardless of the water content, the average diameter of the scattering particles was 0.51 ± 0.03 nm. This was indistinguishable from the solvent mixtures not containing phthalocyanines. We noted that this result merely suggested that large size (>10 nm) aggregates were not present in solutions. Considering the dimensions of the phthalocyanine molecules, the size of dimers and small aggregates would be ca. 1–2 nm (assuming hard spheres). The small aggregates were too small to be reliably detected by the DLS. We also noted that due to the fluorescence of the phthalocyanines, only very dilute solutions (∼1 × 10^–6^ M) could be tested. This complicated the task even further, as the number of aggregates in solutions was likely below the detection limit of the method.

### Adsorption of the Fluorinated Phthalocyanines on Solid Supports

**Figure 6:**
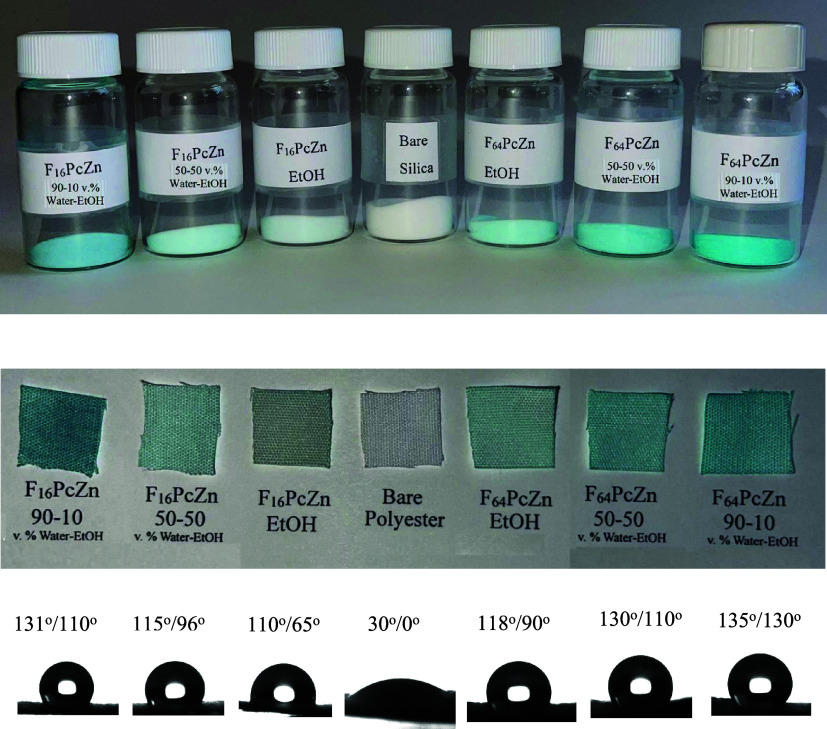
Phthalocyanine-coated silica gels (top) and polyester textiles (middle) obtained by equilibrium adsorption from 100 μM solutions of F_16_ and F_64_PcZn in water–ethanol mixtures of different compositions. Bottom: advancing/receding contact angles and the images of the water drops sitting on the corresponding phthalocyanine-coated polyester textiles.

**Figure 7:**
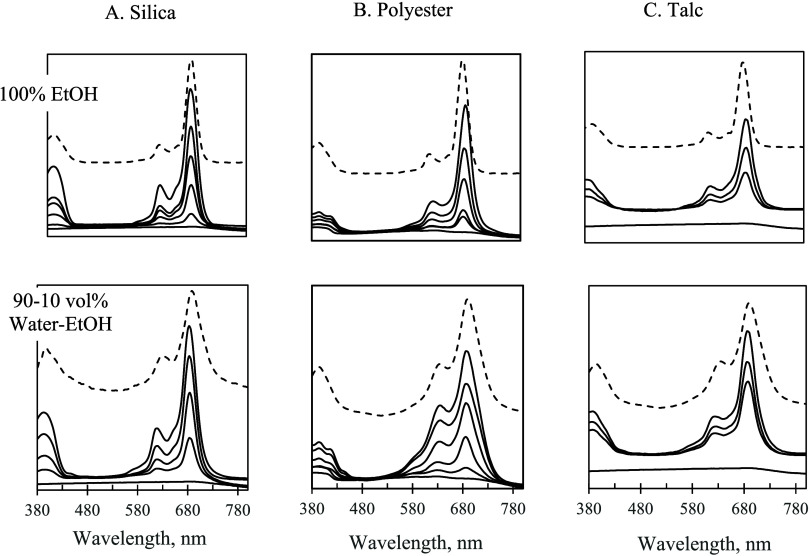
Solid-state UV–vis spectra for F_64_PcZn supported on silica (Column A), polyester (Column B), and talc (Column C). Top row: adsorption from 100% EtOH. Bottom row: adsorption from 90 to 10 vol % water-EtOH. Range of solution concentrations of F_64_PcZn used for the adsorption: 0–200 μM. Solution spectra of F_64_PcZn in the corresponding water–ethanol mixtures are shown with dashed lines (offset for clarity).

**Figure 8:**
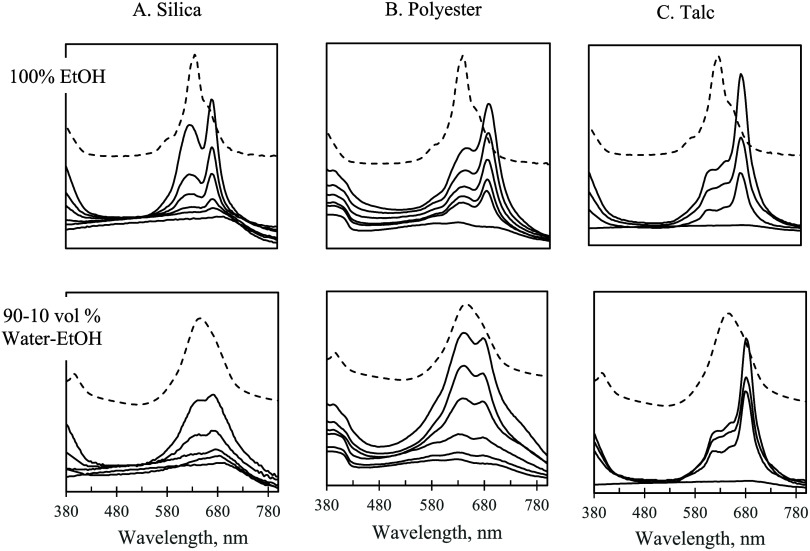
Solid-state UV–vis spectra for F_16_PcZn supported on silica (Column A), polyester (Column B), and talc (Column C). Top row: adsorption from 100% EtOH. Bottom row: adsorption from 90 to 10 vol % water-EtOH. Range of solution concentrations of F_16_PcZn used for adsorption: 0–200 μM. Solution spectra of F_16_PcZn in the corresponding water–ethanol mixtures are shown with dashed lines (offset for clarity).

The adsorption of F_16_PcZn and F_64_PcZn from their water–ethanol solutions was investigated for minerals (silica gel, talc, and mica) and organic polymers (poly(ethylene terephthalate) films and nylon and polyester textiles), which represented two main types of substrates with potential applications in photodynamic surfaces, catalysts, and sensors. For each type of substrate, two different morphologies were studied: planar (PET films and mica sheets) and dispersed (polyester and nylon fibers and particles of talc and silica gel). The planar substrates were well suited for the characterization by surface-sensitive techniques like contact angles and XPS. The dispersed substrates, due to the developed surfaces, were better suited for bulk methods of analysis including the solid-state electronic spectroscopy and the photosensitizing activity of the coatings. The latter was tested through the photo-oxidation of model organic compound in water (methyl orange dye). The visual results of the phthalocyanine adsorption on silica gel and polyester textile are shown in Figure 6. As seen by the change in the substrate color intensity, the phthalocyanines were readily adsorbed on both substrates and the solution composition, i.e., % of water, played a role in the adsorption. The following general pattern was observed for all of the substrates: the phthalocyanine absorption increased with the amount of water present in solutions. The phthalocyanine type, solvent composition, and the substrate nature were important in determining the state of the adsorbed molecules. For the adsorption of F_64_PcZn, the outcome was straightforward and nearly invariant. The solid-state UV–vis demonstrated narrow Q-band at 685 nm, which was indicative of nonaggregated, monomeric F_64_PcZn observed for the adsorption on silica, talc (Figure 7), and nylon (Figure S2). The only exception was the adsorption from 90 to 10 vol % water–ethanol on polyester, where a certain fraction of aggregates were present in the adsorbed state, Figure 7. In the case of F_16_PcZn, as compared to solutions, the fraction of the monomeric phthalocyanines significantly increased in the adsorbed state. This was demonstrated by the monomeric peak at 675–680 nm for the adsorbed F_16_PcZn (Figure 8, top row), which was dominant in the spectra for all of the substrates when the adsorption was carried out from ethanol. However, when the adsorption was carried out from 90 to 10 vol % water–ethanol, the state of the adsorbed F_16_PcZn was strongly influenced by the substrate. The adsorption on talc produced mostly monomeric F_16_PcZn, while the adsorption on silica, polyester (Figure 8), and nylon (Figure S2) produced surfaces with a sizable fraction of aggregates, as evidenced by the growth of the “aggregate” peak at ∼630 nm. Interestingly, these results suggested that the hydrophobicity of the mineral substrates played little to no role in the self-association of F_16_PcZn in the adsorbed state. The adsorption of F_16_PcZn on hydrophobic talc produced monomers, yet its adsorption on hydrophobic polyester and nylon produced aggregates. Trying to access the role of surface OH groups in surface aggregation, we tested the adsorption of F_16_PcZn from 90 to 10 vol % water–ethanol on silicas with different extents of surface hydroxylation. To our surprise, we found not much difference in the UV–vis of F_16_PcZn adsorbed on fully hydroxylated, hydrophilic silica vs severely dehydroxylated, hydrophobic silica obtained via calcination at 800 and 1000 °C, Figure S3. We noted that talc differed substantially from silica and polymers in the sense that, unlike the latter, talc was capable of strong interactions with F_16_PcZn. The adsorption of F_16_PcZn on talc can involve specific interactions between the electron-deficient metal center (Lewis acid) and the basic centers on the surface of talc (Mg–OH). Such specific interactions provided an additional (to dispersive interactions) contribution to the total adsorption energy, which promoted the formation of monomers. The interactions involving electronic states of Zn and N of phthalocyanine (F_16_PcZn) adsorbed on mica, the material chemically related to talc, were supported by XPS (see below). Strong surface coupling involving Zn and N centers of the phthalocyanine was observed previously for titania,^23^ where the adsorbed F_16_PcZn was also present in mostly monomeric form.

**Figure 9:**
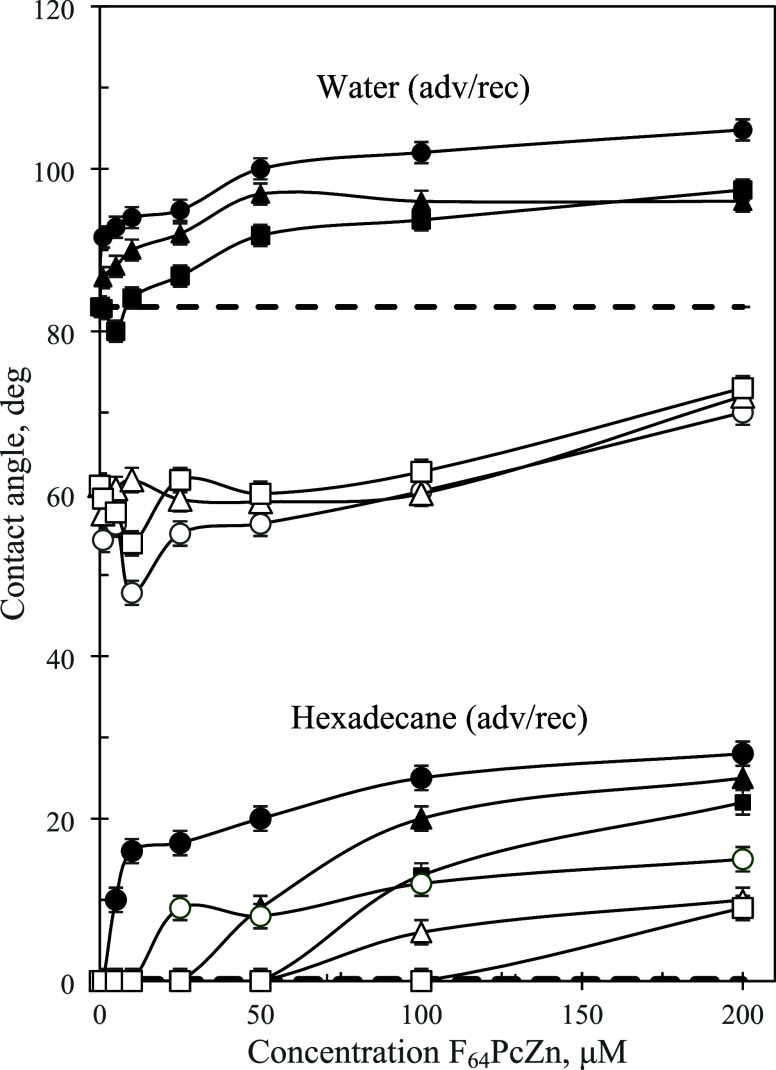
Water and hexadecane contact angles for surfaces of F_64_PcZn adsorbed on PET from water–ethanol mixtures of different composition: (○) 90–10 vol % water–ethanol; (Δ) 50–50 vol % water–ethanol; (□) pure ethanol. Closed and open symbols are for advancing and receding angles, respectively. Dashed lines correspond to the contact angles (adv) for bare PET.

The surface properties, specifically the wetting of the adsorbed phthalocyanines, are discussed next. As a demonstration of wetting interactions, Figure 6 (bottom) presents the contact angles and the images of the water drops sitting on the polyester textiles with the adsorbed phthalocyanines. As evidenced by the contact angles, the adsorption of phthalocyanines on the polyester produced hydrophobic surfaces. The most uniform and the most hydrophobic surfaces were obtained by the adsorption of phthalocyanines from the solutions containing 90 vol % water. Due to low wetting hysteresis, the water drops rolled freely on these surfaces, demonstrating very low adhesion. The adsorption from pure ethanol and from 50−50 vol % water–ethanol mixtures also produced hydrophobic surfaces, yet the receding angles for these surfaces were notably lower, especially for F_16_PcZn. This was attributed to the reduced surface coverage and presence of accessible bare substrate for the surfaces produced by adsorption from solutions with high ethanol content. The poor wetting of the phthalocyanine-coated textiles was in clear contrast with the good wetting of bare polyester, Figure 6. Although the smooth surface of polyester was moderately hydrophobic (water CA 83°/61°, adv/rec),^24^ due to a woven pattern, the polyester textile did not support water droplets as they were rapidly soaked in by capillary forces. For phthalocyanines adsorbed on smooth substrates (PET films and mica), the contact angles were not influenced by the surface roughness and provided more insight into the local surface structures. Figure 9 shows the contact angle data for F_64_PcZn on PET. The data for F_16_PcZn is shown in Figure S4. As shown in Figures 9 and S4, the contact angles increased with the solution concentration of the phthalocyanines, which was consistent with an increase in the phthalocyanine adsorption. The contact angles of hexadecane were particularly informative of this trend since, unlike water, hexadecane completely wetted bare PET (contact angles ∼0°). An increase in the hexadecane contact angle after exposure to phthalocyanine solutions indicated the presence of lyophobic groups (F, and *i-*C_3_F_7_) on the surfaces. Table 2 summarizes the saturation contact angles observed for the phthalocyanines adsorbed on PET and mica from water/ethanol solutions of different compositions.

**Table 2:** Contact Angles for PET and Mica after Exposure to 200 μM Solutions of the Phthalocyanines of Different Water–Ethanol Composition

		contact angles (adv/rec), deg	
Pc and substrate	vol. % water in solution used for adsorption	water	hexadecane
F_16_PcZn_PET	0	97/52	<5
	50	105/58	<5
	90	107/64	7/0
F_16_PcZn_Mica	0	86/49	<5
	50	98/53	<5
	90	99/58	9/0
F_64_PcZn_PET	0	96/73	22/<5
	50	97/72	25/<5
	90	105/61	25/13
F_64_PcZn_Mica	0	93/70	23/9
	50	99/73	28/12
	90	109/60	33/13

As evidenced by the contact angles in Table 2, the most hydro- and oleophobic surfaces were produced by the adsorption of phthalocyanines from the solutions containing the highest amount of water (90 vol %). For further analysis and interpretation of the contact angles provided in Table 2, a proper reference for the contact angles for pure phthalocyanines was needed. As no such data were found in the literature, the surfaces of neat F_16_ and F_64_PcZn supported on smooth substrates (Si-wafers, mica, and glass) were prepared by sublimation, and their contact angles were measured. The details on the preparation and characterization of the sublimed films are provided in the Supplement. The average thickness of these films was ca. 50–150 nm. The contact angles of the sublimed films showed almost no variation with the substrate and thus were characteristic of neat amorphous phthalocyanines (Table S3). For the sublimed films of F_64_PcZn, the average contact angles (advancing/receding) were 113 ± 2/70 ± 3° for water and 54 ± 3/13 ± 2° for hexadecane, respectively. These values were in excellent agreement with 115 and 56°, the water and hexadecane advancing contact angles reported for monolayers of perfluoroisopropyl groups,^25^ that could serve as a good model for the perfluoroisopropyl perimeter of F_64_PcZn. For neat F_16_PcZn, the corresponding water and hexadecane contact angles were 95 ± 1/63 ± 2° (water) and 12 ± 4/0° (hexadecane). These angles were consistent with the monolayers exposing perfluorophenyl groups.^25^ As compared to neat phthalocyanines, the contact angles for the phthalocyanines adsorbed on PET and mica (Table 2) were notably smaller, especially when hexadecane was used as the probe fluid. We considered two main factors that could contribute to that. First, incomplete surface coverage and the presence of bare substrate could be a reason for the lower contact angles observed for the adsorbed films. However, the surface coverage, as assessed from the XPS thickness (see below), was consistent with complete monolayers. So, the low contact angles could not be attributed to low surface coverage. More likely, the difference in wetting of neat films vs adsorbed films was due to differences in the orientation of the phthalocyanine molecules. Films obtained by sublimation were relatively thick (∼10^2^ nm) as compared to monolayers and consisted of randomly oriented phthalocyanine molecules exposing their low-energy groups to minimize the surface energy. This explained why their contact angles were in good agreement with the contact angles for surfaces rich in CF_3_ (F_64_PcZn) and CF (F_16_PcZn) functionalities. However, in the monomolecular surfaces obtained by the adsorption of phthalocyanines from solutions, the orientation of the molecules was likely “face-down” to maximize their adsorption interactions. The polar central portions of the adsorbed molecules in the “face-down” orientation were more accessible for the probe fluids resulting in lower advancing and receding contact angles.

**Figure 10:**
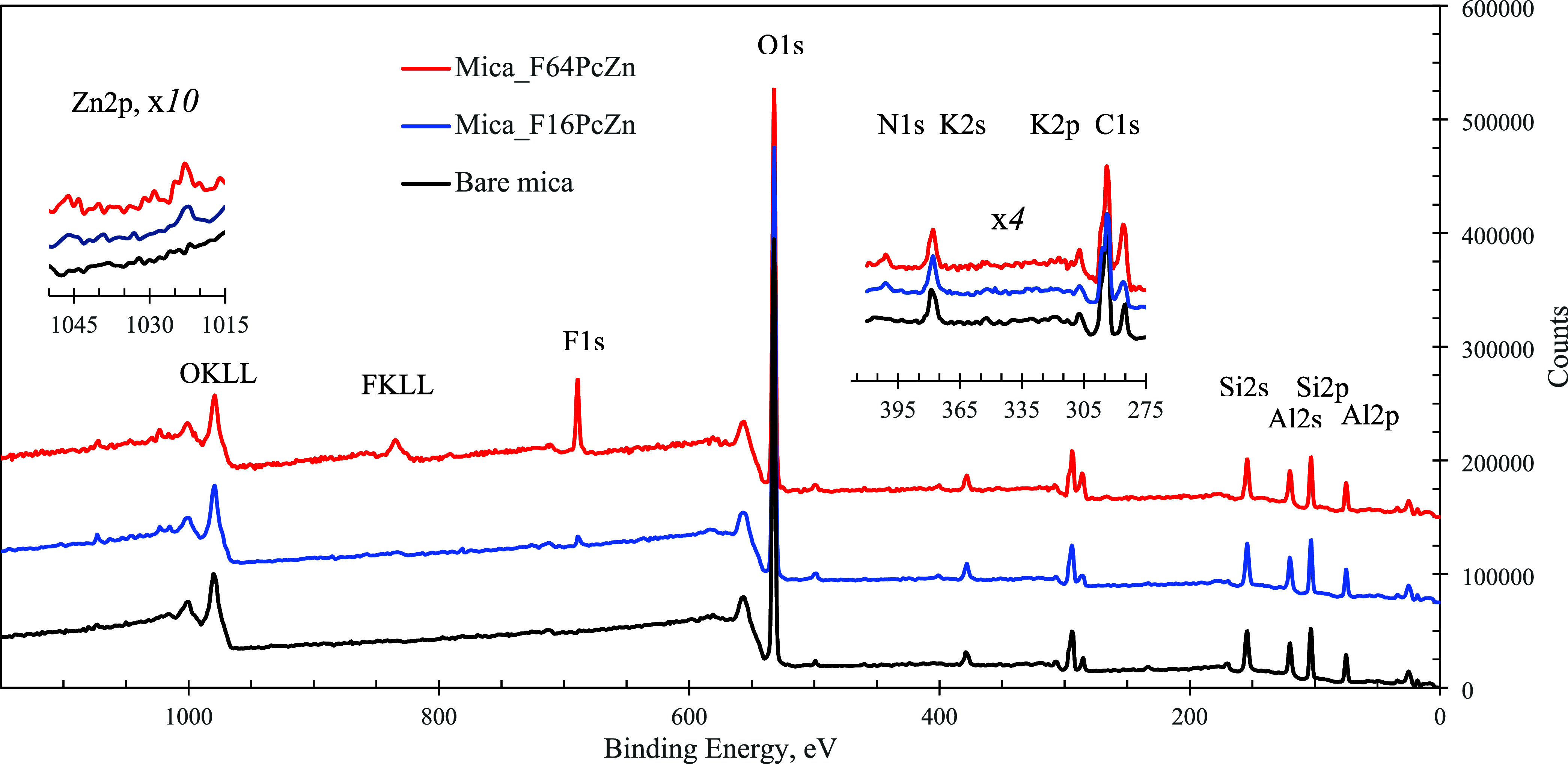
XPS survey spectra of bare mica and mica after contact with 100 μM solutions of F_64_ and F_16_PcZn in 50–50 vol % water–ethanol. Spectra are offset for clarity. The insets present close-ups of Zn 2p and N 1s, K 2s, K 2p, and C 1s regions.

**Figure 11:**
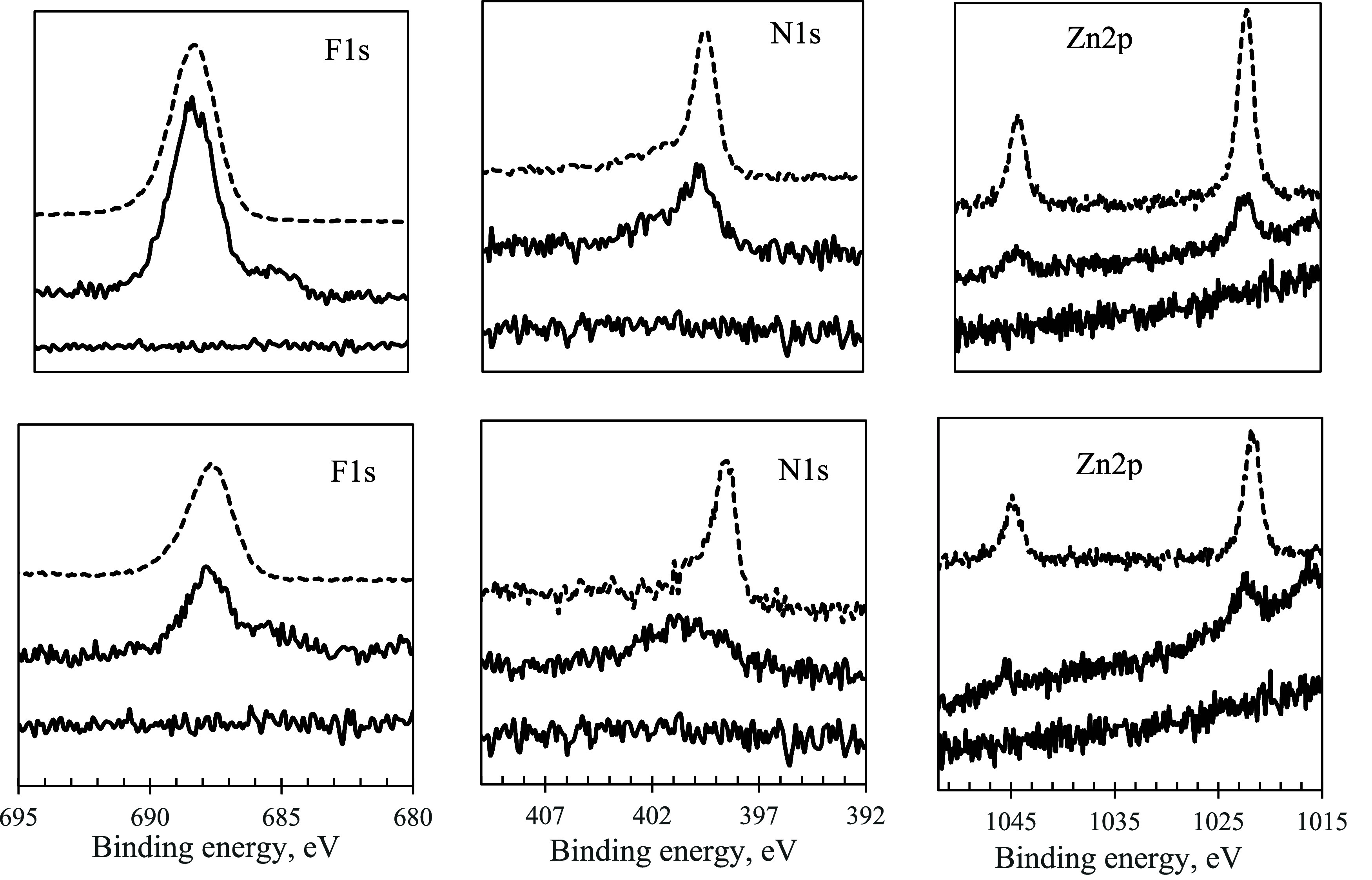
F 1s, N 1s, and Zn 2p XPS core level spectra for bare mica, and for F_64_PcZn (top row) and F_16_PcZn (bottom row) adsorbed on mica. The spectra for bulk Pcs are shown as dashed lines (offset for clarity).

The chemical composition and thickness of the phthalocyanine surfaces adsorbed on mica were characterized by XPS. Figure 10 compares the XPS survey spectra for bare mica and mica that was exposed to the solutions of F_16_PcZn and F_64_PcZn. The samples exposed to the phthalocyanine solutions showed new peaks associated with the elements comprising the phthalocyanine molecules. Specifically, strong peaks at ca. 830–870 and ∼685 eV were attributed to F KLL Auger lines and F 1s photoelectrons, with peaks ∼1045 and 1022 eV to Zn 2p and a peak ∼400 eV to N 1s, respectively. Figure 11 compares the core level spectra of F 1s, N 1s, and Zn 2p for bulk phthalocyanines and phthalocyanines adsorbed on mica. In the F 1s region of the adsorbed phthalocyanines, along with the main peak at ∼688 eV from CF and CF_3_ groups,^23^ an additional smaller peak at ∼685 eV appeared in the spectra. Based on its position, this peak was attributed to the species of fluoride^26^ (HF or F^–^). The fluorides were likely formed as a result of a partial displacement of aromatic fluorine atoms by surface OH groups or by water chemisorbed on unsaturated surface sites. The presence and the intensity of the peak ∼685 eV were not influenced by the amount of water in the solution used for the adsorption, arguing that the hydrolysis of the aromatic fluorines did not occur in the solutions. As verified by UV–vis and MALDI, the integrity of the phthalocyanines remained unchanged for months in the water–ethanol solutions. The metal oxide polar surfaces can play a critical (catalytic) role in the activation of water and partial hydrolysis of the aromatic fluorines of the phthalocyanines. This reaction was reported previously^23^ for F_16_PcZn adsorbed on titania. As evidenced by the relative intensities of the fluoride peaks, the planar F_16_PcZn was more susceptible to hydrolysis (*I*_685_/*I*_688_ ∼ 0.25) as compared to its bulky counterpart F_64_PcZn (*I*_685_/*I*_688_ ∼ 0.12). N 1s spectra of the adsorbed F_16_PcZn were broadened, and their binding energy was shifted to higher values by ∼2 eV vs bulk. Such a major change indicated the involvement of nitrogen atoms in the interactions with the surface that changes their chemical environment, likely via protonation by the surface hydroxyl groups.^27^ For the adsorbed F_16_PcZn, the Zn 2p_3/2_ band was shifted up by 0.6 eV vs bulk (from 1021.8 to 1022.4 eV, respectively). This was also attributed to the coordination and/or H-bonding type interactions of Zn^2+^ with the surface hydroxyls.^23,28^ In the case of F_64_PcZn, H-bonding between the macrocycle and the surface was not possible for steric reasons. N 1s and Zn 2p core level spectra for the adsorbed and bulk F_64_PcZn were quite similar, indicating that these sites of the molecules were not perturbed by the interaction with the surface. O 1s, Si 2p, Al 2p, and K 2p core level spectra of the adsorbed phthalocyanines were similar to those of bare mica. Only the intensities of these signals decreased due to attenuation by the adsorbed films. The attenuation of the most prominent O 1s peak was used to calculate the average thickness of the adsorbed films using the equation:^29^
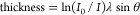
where *I*_0_ and *I* are the intensity of the O 1s peak (532.2 eV) for bare mica and mica with the adsorbed PcM, respectively; θ *=* 90° is the XPS takeoff angle; and λ = 2.8 nm, the O 1s photoelectron mean free path for mica.^30^ The results are summarized in Table 3. The values of thickness were in the range of tenths of nm. The highest values of thickness were obtained for the adsorption from the solutions containing 90 vol % water: 0.3 ± 0.1 nm for F_16_PcZn and 0.5 ± 0.1 nm for F_64_PcZn, respectively. These values were close to the thickness of the phthalocyanine molecules^23^ 0.33 for F_16_PcZn and 0.61 nm for F_64_PcZn supporting the “face-down” orientation of the phthalocyanines on the surface.

**Table 3:** XPS Thickness of the Phthalocyanine Layers Supported on Mica

	**F**_**64**_**PcZn**	**F**_**16**_**PcZn**
**solution used for adsorption**^a^	**thickness, nm**	
EtOH	0.3 ± 0.1	0.2 ± 0.1
90–10 vol % water-EtOH	0.5 ± 0.1	0.3 ± 0.1

a Phthalocyanine concentration: 100 μM.

### Photodynamic Activity of the Fluorinated Phthalocyanine Coatings

**Figure 12:**
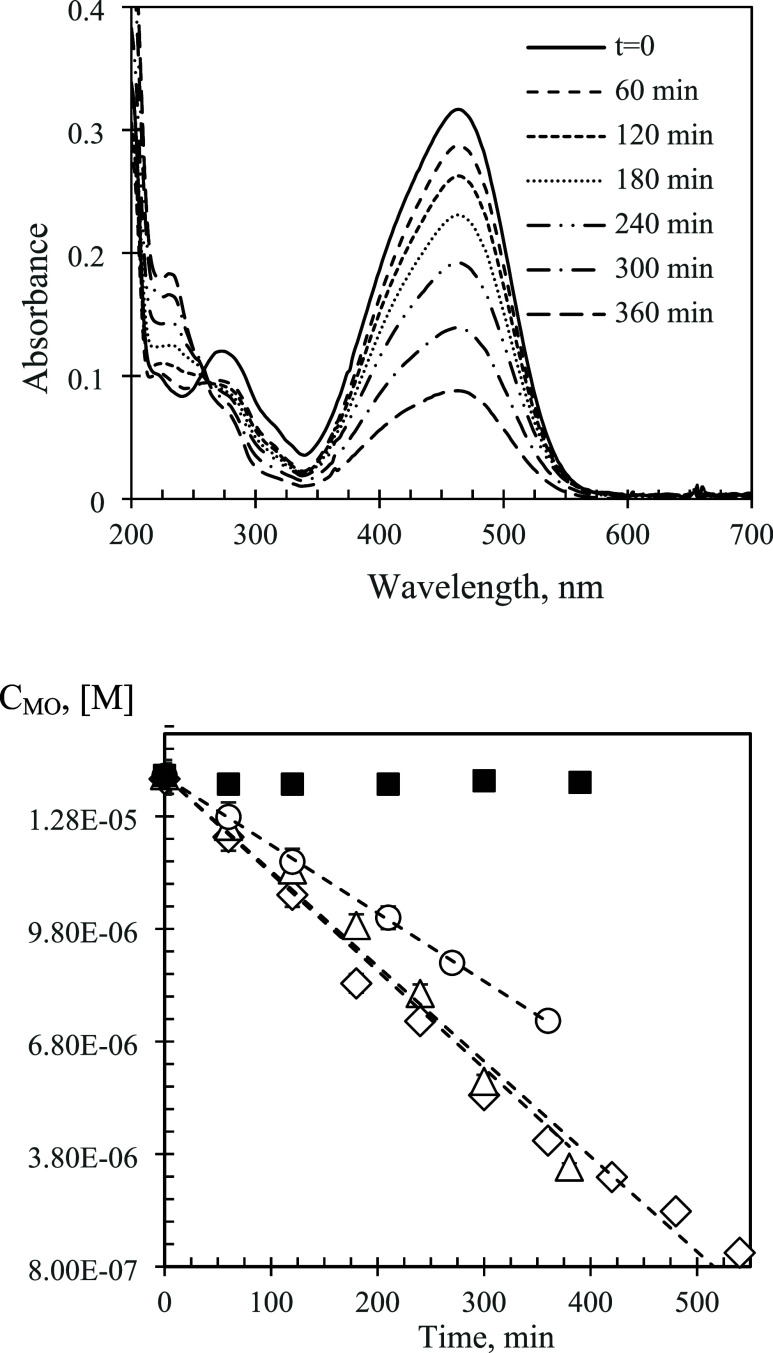
Top: UV–vis spectra of methyl orange (MO) solutions taken at different radiation times. Bottom: Kinetic plots for MO degradation by F_16_PcZn supported on polyester. The phthalocyanine was adsorbed from its solutions in 90–10 vol % Water-EtOH (◊); 50–50 vol % Water-EtOH (Δ); and 100% EtOH (○). Solid symbols – essentially no degradation was observed in the dark. Trendlines correspond to zeroth order kinetic fits.

The activity of the phthalocyanine coatings supported on polyester textile and talc was tested in the reaction of degradation of methyl orange dye (MO) under visible light radiation. MO was chosen as a model water pollutant,^31^ whose degradation can be easily monitored by the disappearance of its absorption at 464 nm via UV–vis spectroscopy. The mechanism^32^ of photodegradation of MO by the phthalocyanine-based photosensitizers involved the production of singlet oxygen and other reactive oxygen species (ROS) such as HOO^•^, and HO^•^. No reaction occurred in the absence of light or under an inert atmosphere (Nitrogen). The adsorption/degradation of MO on/by bare substrates (no phthalocyanines) was also negligible as no changes in the UV–vis of MO in solution were observed after 4 h of contact (Figure S5). The solid-state spectra of the catalysts showed virtually no changes (no photobleaching) before and after the catalysis runs; also, no phthalocyanine was detected in the solution phase (UV–vis), demonstrating the stability of the photocatalysts. The representative photodegradation time plots are shown in Figure 12. All of the kinetics plots were fitted using the zeroth order coordinates. The corresponding rate constants are summarized in Table 4.

**Table 4:** Kinetics of the Methyl Orange Photodegradation by the Solid-Supported Phthalocyanines

catalyst	phthalocyanine loading (±0.1),^a^ μmol/g	rate constant (±0.2), 10^–8^ M/min
F_16_PcZn_Talc	1.5	4.1
F_64_PcZn_Talc	1.0	4.5
F_16_PcZn_Polyester	1.0	2.8
F_64_PcZn_Polyester	0.5	2.6

a All coatings were prepared using 100 μM solutions of phthalocyanines in 90–10 vol % Water-EtOH.

According to the rate constants, the most active surfaces were obtained for F_64_PcZn supported on talc. For the phthalocyanines supported on polyester, the rate constants were ∼1.7 times smaller than those on talc, which was attributed to a lower loading of the phthalocyanines. Interestingly, the surfaces of F_16_PcZn and F_64_PcZn (on talc and on polyester) demonstrated very similar activity. This observation was of particular importance with regard to the monomer–aggregate equilibrium of the adsorbed phthalocyanines (Figures 7 and 8 and discussion above). According to the literature,^33^ the aggregation of phthalocyanines decreases the lifetime of the excited states and lowers the generation of singlet oxygen, thereby reducing their activity in photocatalysis. In aqueous solutions, F_64_PcZn as well as F_16_PcZn were aggregated. In the adsorbed state, however, as a result of the adsorption interactions, the self-association equilibrium was shifted toward monomers. For the strong adsorbing talc, the surfaces of F_16_PcZn and F_64_PcZn were mostly monomeric, which explained their high activity. For polyester, where the adsorption was governed by weak dispersive interactions, the aggregates were present. The fraction of the aggregates (peak ∼ 630 nm) grew for the surfaces prepared by the adsorption from the solutions with high water content, as shown in Figure 7. While only the direct measurements can unequivocally quantify the intricate role of aggregation on the production of singlet oxygen by these surfaces, here, we can safely conclude that although some aggregates were present in the case of the adsorbed F_16_PcZn, its activity was sufficient to degrade MO at the same rate as F_64_PcZn.

## Conclusions

We reported the preparation and characterization of aqueous solutions of perfluorinated phthalocyanine photosensitizers F*_x_*PcZn (*x* = 16, 64). The unexpectedly high amount of water (up to 98 vol %) that can be used as a cosolvent for these phthalocyanines was attributed to the amphiphilic nature of the macrocycles, in which the highly polar metal porphyrin center was surrounded by electron-withdrawing nonpolar groups (F and CF_3_). A thermodynamic model of interfacial interactions in solutions of amphiphilic phthalocyanines was proposed, explaining their solubility in water–ethanol mixtures. As assessed by UV–vis and fluorescence, F_16_ and F_64_PcZn in aqueous solutions were aggregated; however, the size of these aggregates was small and not detectable by DLS. Aqueous solutions of the phthalocyanines were used to prepare coatings on a variety of substrates, including PET plastic, polyester and nylon textiles, mica, silica gel, and talc. According to XPS, solid-state UV–vis, and contact angles, the equilibrium solution adsorption produced hydrophobic, ∼monomolecular coatings of the phthalocyanines. In the adsorbed state, the self-association equilibrium was shifted toward monomeric, nonaggregated form of the phthalocyanines. The photodynamic activity of the solid-supported phthalocyanines was tested in the reactions of degradation of model organic contaminant (methyl orange dye) under radiation with visible light. Both F_16_PcZn and F_64_PcZn produced stable and reproducible coatings capable of generating reactive oxygen species that degraded the methyl orange dye in water.

## Supporting Information Available

Details on thermodynamic model of interfacial interactions of fluorinated zinc phthalocyanines in water–ethanol and the results of the calculations (Tables S1 and S2); fluorescence spectra of F_64_PcZn in aqueous solutions (Figure S1); solid-state UV–vis spectra of the phthalocyanines supported on nylon and calcinated silica (Figures S2 and S3); experimental details on the preparation and wetting of the sublimed phthalocyanine films (Table S1); water contact angles for F_16_PcZn supported on PET (Figure S4); and UV–vis spectra of MO solutions in contact with bare polyester and with F_64_PcZn_polyester in the dark (Figure S5) (PDF)
