# Supplementary material for: The Unexpected High Solubility of Fluorinated Zinc Phthalocyanines in Aqueous Solutions and Their Use for the Preparation of Photodynamic Coatings on Various Substrates
Source: Langmuir. 2025 Mar 20;41(12):8202–13. doi: 10.1021/acs.langmuir.4c05325 (PMC11966781; doi:10.1021/acs.langmuir.4c05325)

# Supplement data

to "The Unexpected High Solubility of Fluorinated Zinc Phthalocyanines in Aqueous Solutions and Their Use for the Preparation of Photodynamic Coatings on Various Substrates"

by Jonathan Pinnock[1], Kai Hansen[1], Marius Pelmuş[1,2], and Alexander Y. Fadeev[1*]

[1]*Department of Chemistry and Biochemistry, Seton Hall University, South Orange, NJ 07079, USA*

[2]*Center for Functional Materials, Seton Hall University, South Orange, NJ 07079, USA*

Email: alexander.fadeev@shu.edu

Number of pages: 11

Number of figures: 5

Number of schemes: n/a

Number of tables: 3

TOC Graph

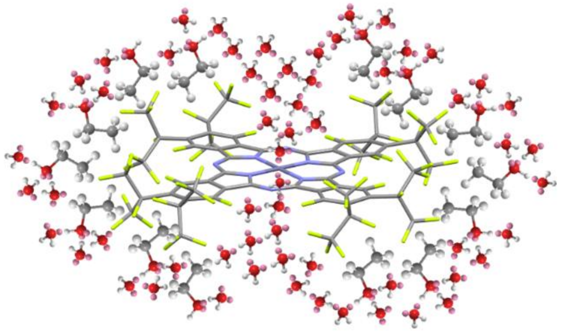

*On the thermodynamic model of interactions between FxPcZn and water-ethanol.*

Molecular areas of the phthalocyanine molecules were determined for the structures optimized in ChemDraw using the software MarvinView, Version 24.1.2 (ChemAxon Ltd., 2024) on Windows 10, with the option Tools\Geometry\Geometrical Descriptors, Table S1.

**Table S1**

Area of the phthalocyanine molecules

| | **$F_{16}PcZn$** | **$F_{64}PcZn$** |
|---|---|---|
| Van der Waals surface, $nm^2$/molecule | 5.33 | 11.21 |
| Van der Waals surface, $m^2/g$ | 3706 | 3267 |
| Polar surface $A_{polar}$, $nm^2$/molecule | 1.86 | 1.86 |
| Nonpolar surface $A_{nonpolar}$, $nm^2$/molecule | 3.47 | 9.35 |

According to[1], an increase in free energy due to insertion of a nonpolar (hydrophobic) molecule in water is

$$\Delta G_{nonpolar} = \gamma_{sl} \cdot A_{nonpolar} \cdot N_A > 0 \qquad \text{(S1)}$$

where $\gamma_{sl}$ - interfacial tension at water-hydrophobic interface ($J/m^2$), $A_{nonpolar}$ - molecular area ($m^2$), and $N_A$ - Avogadro's number ($mole^{-1}$). The values of $\gamma_{sl}$ for the nonpolar portion of the molecules were calculated via Young's equation (eq.S2) using the literature data on the surface tension $\gamma_{lv}$ and the contact angles $\theta$ for water-ethanol on poly(tetrafluoroethylene) (PTFE).[2,3]

$$\gamma_{sl} = \gamma_{sv} - \gamma_{lv} \cdot cos\theta \qquad \text{(S2)}$$

$\gamma_{sv}$ was 19 $mJ/m^2$, the surface energy of PTFE.[4] The data from the refs 2,3 and the results of the calculations by eqns. S1 and S2 are presented in Table S2. As expected, $\Delta G_{nonplar}$ decreased for

the solutions with the higher ethanol content, yet it remained positive indicating an unfavorable process of creation of a lyophobic interface. For the polar portion of the molecule ($A_{polar}$), interfacial tension $\gamma_{sl}$ was assumed to be zero.

For spontaneous dissolution of the phthalocyanines, an increase in free energy due to the formation of a lyophobic interface $\Delta G_{nonpolar} > 0$ must be compensated by the free energy of the interactions of water with the polar center of the molecules $\Delta G_{polar} < 0$ . These interactions included hydrogen bonding/coordination of water with the electron deficient metal center (Lewis acid). This term was calculated as

$$\Delta G_{polar} = -\Delta G_{immersion} \cdot A_{polar} \cdot N_A \qquad \text{(S3)}$$

where $\Delta G_{immersion}$ – literature data on free energy of immersion ($J/m^2$) of polar mineral surfaces, which ranged from ~300 $mJ/m^2$ for water to ~150 $mJ/m^2$ for ethanol.[5] As a lower estimate, we used an average of these values (225 $mJ/m^2$) for all the solutions. This gave -251 kJ/mol for $\Delta G_{polar}$ for both $F_{16}$ and $F_{64}PcZn$ since their $A_{polar}$ were the same. By absolute value $\Delta G_{polar}$ was greater than $\Delta G_{nonpolar}$ and $\Delta G_{net} = \Delta G_{nonpolar} + \Delta G_{polar}$ was negative. Negative values of $\Delta G_{net}$ over the entire range of solution compositions studied demonstrated that the gain from the specific interactions between water and the polar center of the molecules compensated the free energy loss due to formation of a hydrophobic interface, i.e. that dissolution was favorable.

**Table S2**

Interfacial tension and excess free energy at phthalocyanine-water-ethanol interfaces at RT

| | Water-ethanol solution, vol. % | | | |
|---|---|---|---|---|
| | 50-50 | 75-25 | 90-10 | 95-5 |
| Surface tension[2] $\gamma_{lv}$, mJ/m$^2$ | 28 | 38 | 48 | 55 |
| Contact angle[3] on PTFE, deg | 55 | 65 | 80 | 90 |
| Interfacial tension $\gamma_{sl}$ (eq.2), mJ/m$^2$ | 3 | 5 | 11 | 19 |
| $\Delta G_{nonpolar}$ $F_{16}$PcZn (eq.1), kJ/mol | 6 | 10 | 22 | 40 |
| $\Delta G_{nonpolar}$ $F_{64}$PcZn (eq.1), kJ/mol | 16 | 27 | 60 | 107 |
| $\Delta G_{polar}$ same for $F_{16}$ and $F_{64}$PcZn (eq.3), kJ/mol | -251 | -251 | -251 | -251 |

References:


1. Israelashvili, J.N. Intermolecular and Surface Forces. 2d Edition, Academic Press, Amsterdam-Tokyo **1992**.
2. Vazquez, G.; Alvares, E.; Navaza, J.M. Surface Tension of Alcohol + Water at 20-50ºC. *J. Chem. Eng. Data*, **1995**, *40*, 611-614.
3. Tugba Ozturk, T.; Erbil, H.Y. Evaporation of Water-Ethanol Binary Sessile Drop on Fluoropolymer Surfaces: Influence of Relative Humidity. *Colloids and Surfaces A*, **2018**, *553*, 327-336.
4. Spelt, J.K., Moy, E., Kwok, D.Y., Neumann, A.W. The Theory of Surface Tension Components and the Equation of State Approach (p.293). In: Neumann, A.W. and Spelt, J.K. Eds. Applied Surface Thermodynamics. Marcel Dekker, NY, **1996**.
5. Brooks, C.S. Free Energy of Immersion of Clay Minerals in Water, Ethanol, and n-Heptane. *J. Phys. Chem.*, **1960**, *64(5)*, 532-537.

**Figure S1.** From left to right: fluorescence spectra of $F_{64}PcZn$ solutions in pure ethanol, 80-20, and 30-70 vol% ethanol-water (solid lines). Spectra of the solutions spiked with Tween 20 are shown with dashed lines. The excitation wavelength 650 nm.

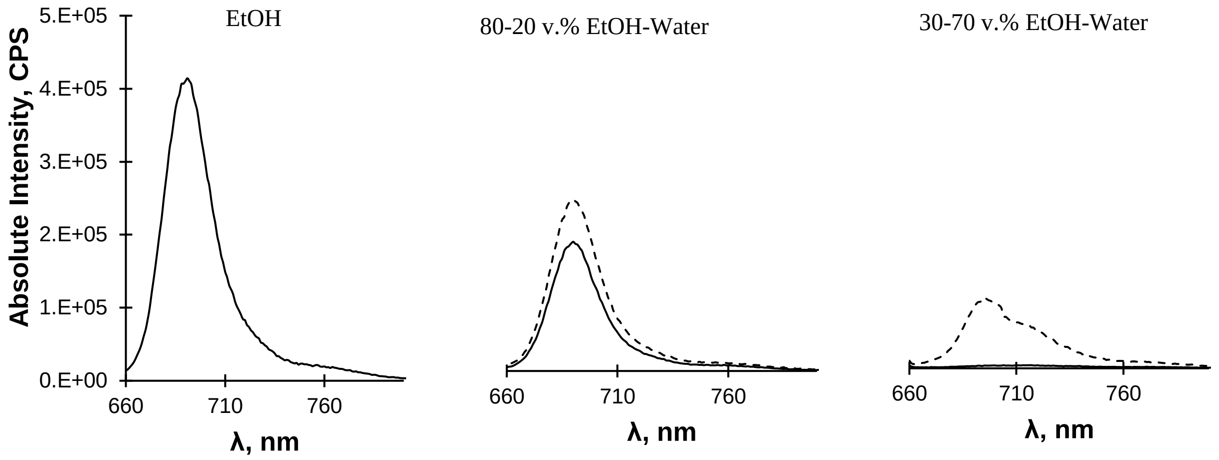

*Phthalocyanine films by vacuum sublimation.* The films were prepared by sublimation of phthalocyanine powders in vacuum (~5 mtorr). The films were deposited on four different substrates: mica, Si wafers, Au-coated Si wafers, and microscope glass slides. Temperature of the sublimation was ~450-500$^{o}$C for $F_{16}$PcZn and ~250$^{o}$C for $F_{64}$PcZn. Temperature of the substrates was RT. The amount of phthalocyanine deposited was determined by dissolution of the deposited films in ethanol and measuring their solution concentrations by UV-Vis. Average thickness of the films was calculated using 1.8 and 1.5 g/cm$^3$ for densities of $F_{16}$ and $F_{64}$PcZn respectively. The thickness was in the range 50-150 nm. The contact angles for the sublimed films are summarized in Table S3.

**Table S3**

Contact angles for the sublimed films of $F_{16}$ and $F_{64}$PcZn

| Phthalocyanine_substrate | Contact angle (adv/rec), deg | |
|---|---|---|
| | Water | Hexadecane |
| $F_{16}$PcZn_glass | 95±1/65±2 | 12±3/0 |
| $F_{16}$PcZn_mica | 96±1/60±2 | 10±5/0 |
| $F_{16}$PcZn_Si wafer | 95±1/62±2 | 10±4/0 |
| $F_{16}$PcZn_Au-Si wafer | 98±1/61±2 | 12±3/0 |
| $F_{64}$PcZn_glass | 115±2/65±5 | 55±3/10±3 |
| $F_{64}$PcZn_mica | 113±2/75±3 | 52±3/15±3 |
| $F_{64}$PcZn_Si wafer | 112±2/73±4 | 55±3/12±2 |
| $F_{64}$PcZn_Au-Si wafer | 115±2/67±3 | 57±3/12±2 |

**Figure S2.** Solid-state UV-Vis spectra for $F_{16}PcZn$ (left column) and $F_{64}PcZn$ (right) supported on nylon. Top row – adsorption from 100% EtOH. Bottom row – adsorption from 90-10 vol% water-EtOH. Range of phthalocyanines solution concentrations used for the adsorption: 0-200 μM. Solution spectra of phthalocyanines in the corresponding water-ethanol mixtures are shown with dashed lines (offset for clarity).

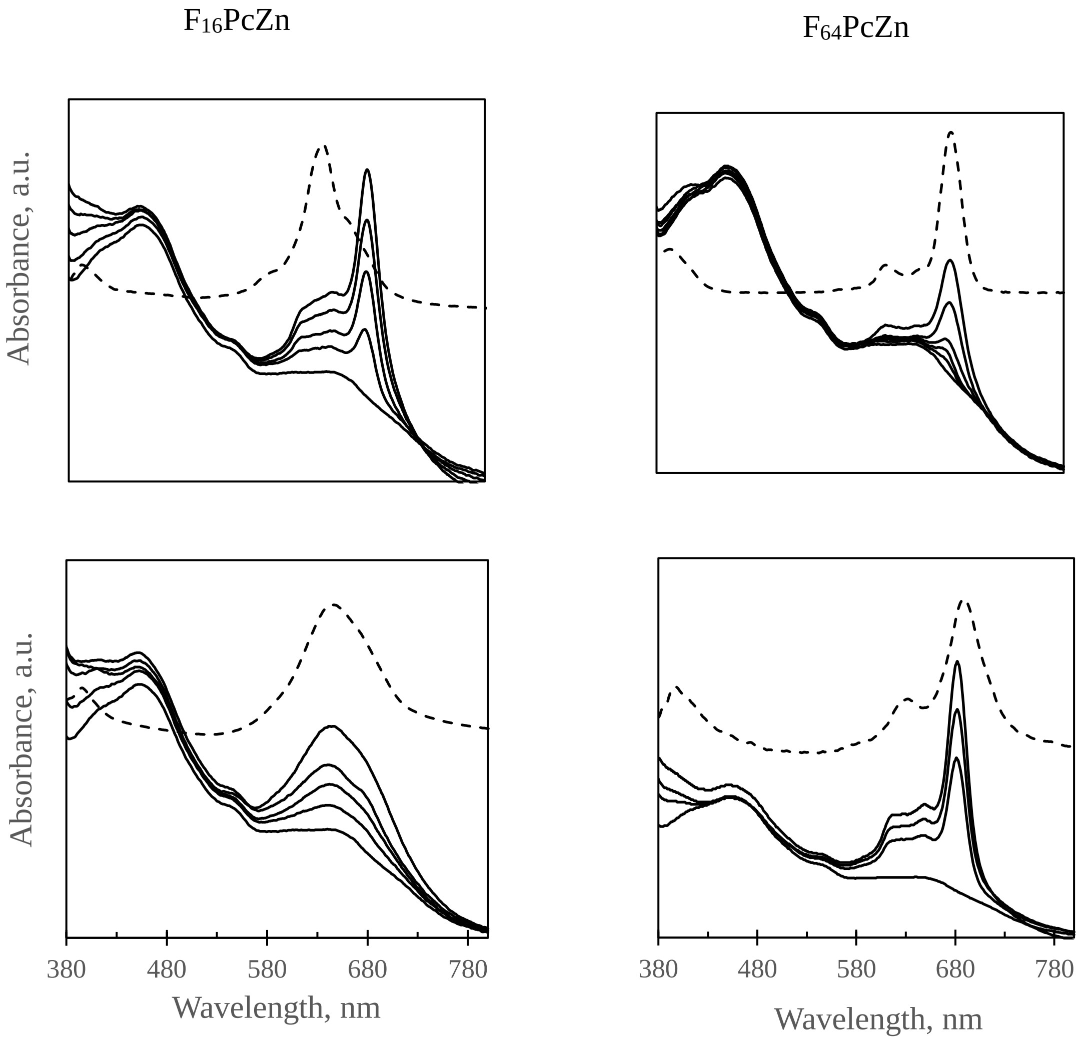

**Figure S3.** Solid-state UV-Vis spectra for $F_{16}PcZn$ adsorbed from 100 μM solutions in 90-10 vol% water-ethanol on silicas treated at different temperatures: *1* – RT (as is), *2* – calcined at 800°C, *3* – calcined at 1000°C. Solution UV-Vis spectra of $F_{16}PcZn$ in ethanol is shown in solid line (offset).

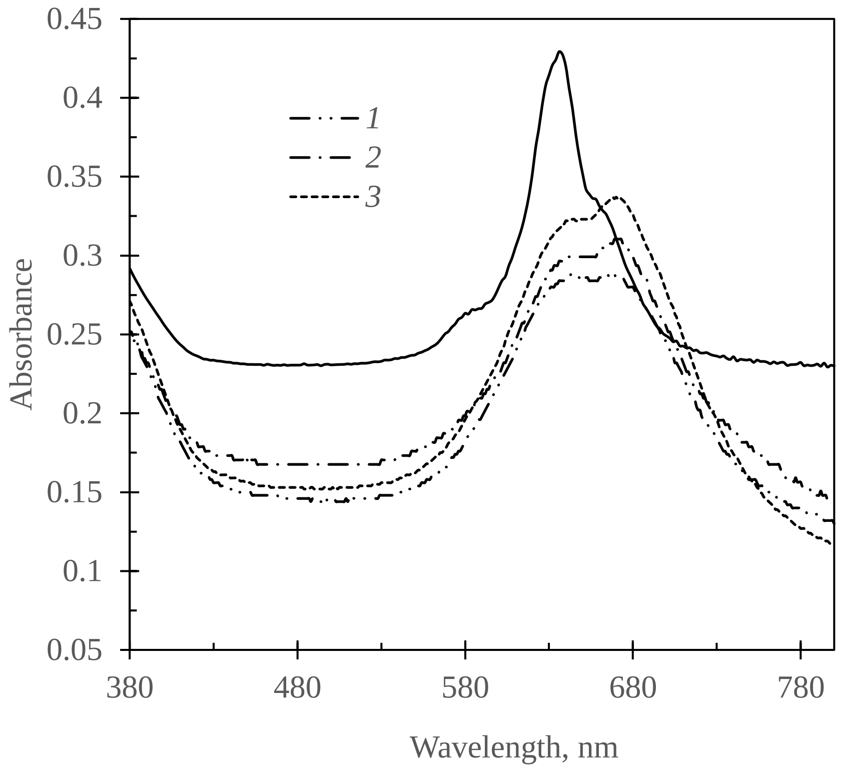

**Figure S4.** Water contact angles for surfaces of $F_{16}$PcZn adsorbed on PET from water-ethanol mixtures of different composition: (○) 90-10 vol% water-ethanol; (Δ) 50-50 vol% water-ethanol; (□) pure ethanol. Closed and open symbols are for advancing and receding angles respectively. Dashed line correspond to the contact angles (adv) for bare PET.

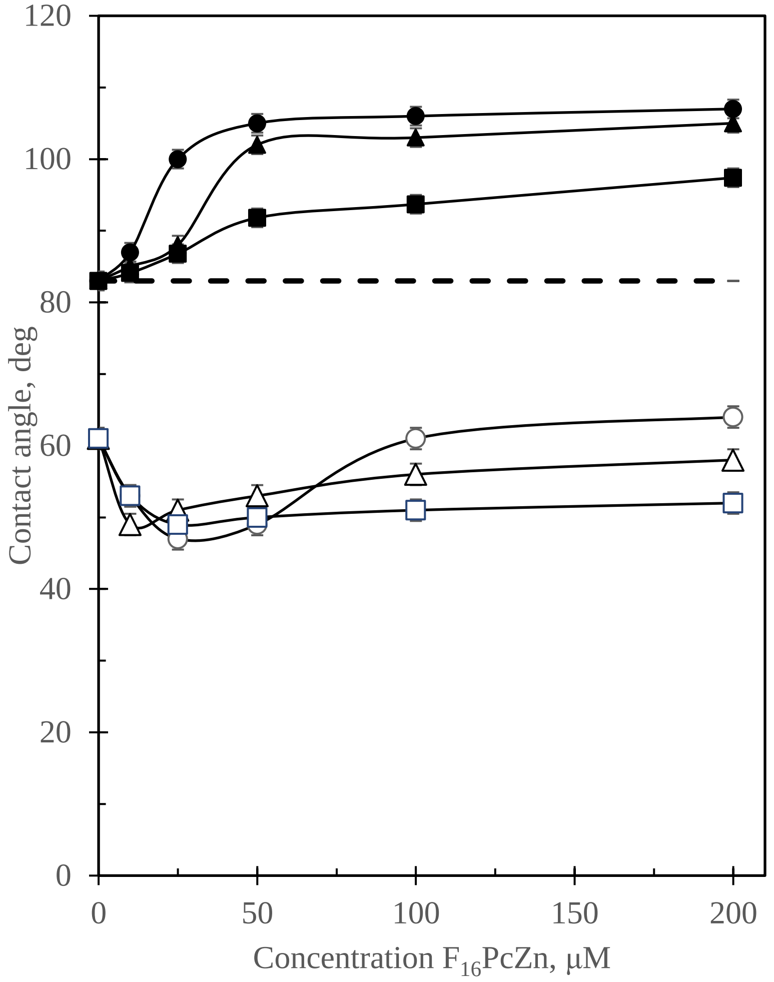

**Figure S5.** Top: UV-vis spectra of methyl orange (MO) solutions in contact with bare polyester (no phthalocyanine) taken at different radiation times. Bottom: UV-vis spectra of MO solutions in contact with $F_{64}$PcZn_polyester in the dark.

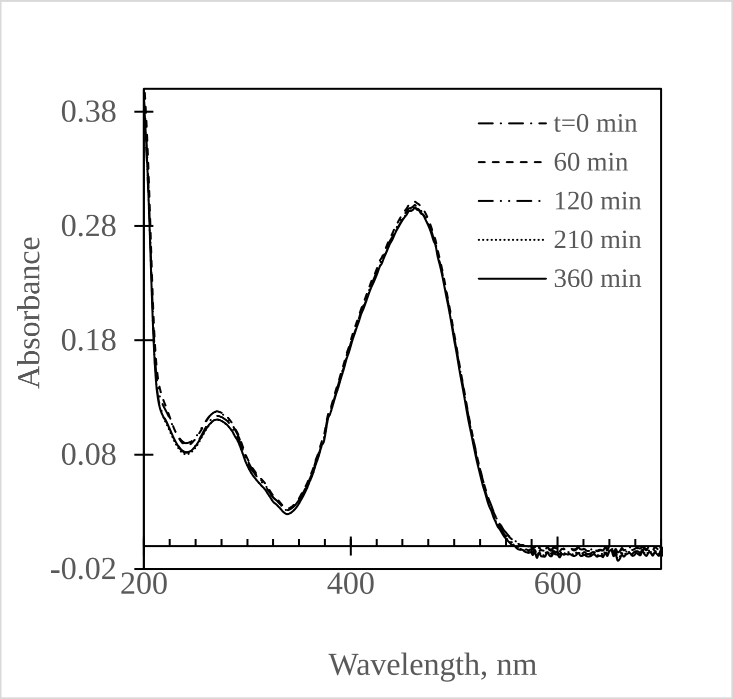


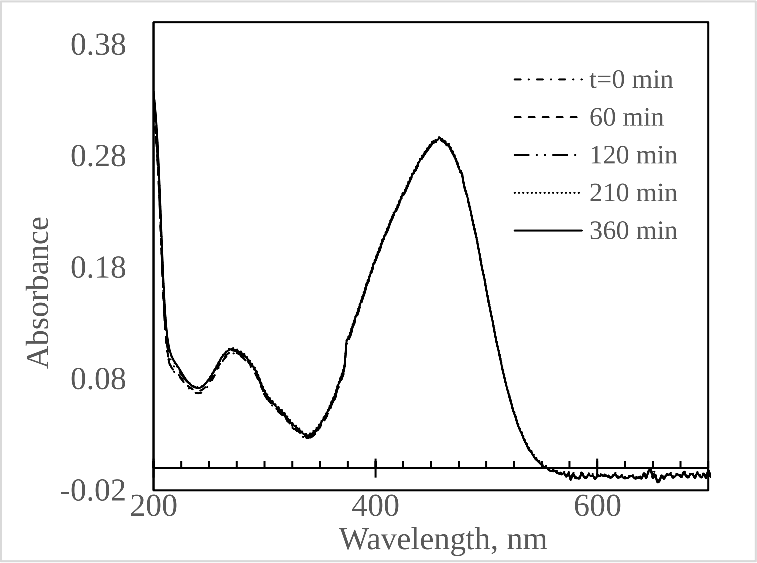

Supplement: Supplementary file 1 — la4c05325_si_001.pdf [file la4c05325_si_001.pdf]
